# Acceleration of protein folding by four orders of magnitude through a single amino acid substitution

**DOI:** 10.1038/srep11840

**Published:** 2015-06-30

**Authors:** Daniel J. A. Roderer, Martin A. Schärer, Marina Rubini, Rudi Glockshuber

**Affiliations:** 1 ETH Zurich, Institute of Molecular Biology and Biophysics, Otto-Stern-Weg 5, CH-8093 Zurich, Switzerland; 2 University of Konstanz, Department of Organic Chemistry, Universitätsstrasse 10, D-78464 Konstanz, Germany

## Abstract

*Cis* prolyl peptide bonds are conserved structural elements in numerous protein families, although their formation is energetically unfavorable, intrinsically slow and often rate-limiting for folding. Here we investigate the reasons underlying the conservation of the *cis* proline that is diagnostic for the fold of thioredoxin-like thiol-disulfide oxidoreductases. We show that replacement of the conserved *cis* proline in thioredoxin by alanine can accelerate spontaneous folding to the native, thermodynamically most stable state by more than four orders of magnitude. However, the resulting *trans* alanine bond leads to small structural rearrangements around the active site that impair the function of thioredoxin as catalyst of electron transfer reactions by more than 100-fold. Our data provide evidence for the absence of a strong evolutionary pressure to achieve intrinsically fast folding rates, which is most likely a consequence of proline isomerases and molecular chaperones that guarantee high *in vivo* folding rates and yields.

The *cis*/*trans* isomerization of prolyl peptide bonds is an intrinsically slow reaction and generally rate-limiting for *in vitro* refolding of proteins harboring *cis* prolyl peptide bonds in their native, three-dimensional structure^1^. About 6% of the X-Pro peptide bonds in high-resolution X-ray structures of proteins adopt the *cis* conformation, while *cis* is only identified in about 0.04% of the non-prolyl peptide bonds^2^. The relatively high frequency of *cis* X-Pro peptide bonds in protein structures can be explained by the fact that the *cis* conformer is only about 5 kJ mol^−1^ less stable than the *trans* conformer^3^. In chemically denatured proteins, this low energy difference is also responsible for the accumulation of 5–30% nonnative *cis* X-Pro peptide bonds at prolines that are otherwise in *trans* in the native state (N)^3^. This leads to the artificial accumulation of slow-folding molecules in *in vitro* protein folding studies in which refolding is initiated by rapid dilution from high to low denaturant concentration, and can significantly complicate the analysis of protein folding kinetics^4^.

The goal of the present study was to establish a model system for studying the coupling between the *trans*-to-*cis* isomerization of a X-Pro peptide bond and conformational folding independently of *in vitro* folding artifacts caused by multiple, nonnative prolyl peptide bonds accumulating in the unfolded state (U). In addition, we intended to find the reasons underlying the strict conservation of *cis* prolyl peptide bonds in certain protein folds. As a model system, we selected *Escherichia coli* thioredoxin (Trx), the prototype of a disulfide oxidoreductase family that is found in all three domains of life^5^,^6^. *E. coli* Trx (108 residues) acts as a cytoplasmic reductant of multiple substrates, including ribonucleotide reductase^7^, methionine sulfoxide reductase^8^, PAPS reductase^9^, peroxidoredoxins^10^,^11^, and the transmembrane reductase DsbD^12^, and receives reducing equivalents from NADPH via thioredoxin reductase. The conserved Trx fold (Fig. 1a) consists of a four-stranded β-sheet flanked by three α-helices^13^,^14^, a catalytic cysteine pair (Cys32 and Cys35) at the N-terminal end of the second α-helix, and an invariant *cis* prolyl peptide bond (Ile75-Pro76) that is buried and located next to the active-site cysteine pair (residue numbering of *E. coli* Trx)^15^.

Wild type (WT) Trx shows a complex *in vitro* folding mechanism with multiple kinetic intermediates^16^ that is dominated by a rate-limiting *trans*-to-*cis* isomerization of the Ile75-Pro76 peptide bond^17^. The folding kinetics are additionally complicated by the four *trans* prolines (residues 34, 40, 64, and 68) of Trx (Fig. 1a) that partially accumulate a non-native *cis* conformation in U. The results indicated that denatured Trx is a mixture of three kinetically distinct, unfolded species (U_VR_, U_R_, and U_M_) that differ in the conformation of the five prolines and collapse to structured intermediates (I_VR_, I_R_, and I_M_) within the dead time (3 ms) of stopped-flow mixing (Fig. 2). The minor species U_VR_ possesses all five prolines in the native conformation, collapses to I_VR_ and very rapidly reaches N. The two major unfolded species U_R_ and U_M_ have Pro76 in the nonnative *trans* conformation. One of these species likely represents molecules with all five prolines in *trans*, while the other corresponds to unfolded molecules with Pro76 in *trans* and one of the other four prolines in the nonnative *cis* conformation. Both intermediates I_M_ and I_R_ react to the intermediate I_S_ with all prolines in the *trans* conformation, and the slow reaction of I_S_ to N (half-live: 280 s) involves the *trans*-to-*cis* isomerization of Pro76 as the common rate-limiting folding step for the majority of Trx molecules (Fig. 2). To study the coupling between folding and the *trans*-to-*cis* isomerization of a single X-Pro peptide bond in a best-defined experimental set-up, we used the single-proline variant Trx1P, in which the four *trans* prolines of Trx were replaced by alanines. Trx1P shows WT-like reductase reactivity, and its X-ray structure is virtually identical to that of Trx WT, with the single Pro76 in *cis*^18^. We show that 95% of the Trx1P molecules accumulate a highly stable intermediate (I_trans_) with Pro76 in *trans*, which reaches N only extremely slowly. Replacement of Pro76 by alanine accelerated folding of the resulting, proline-free variant Trx0P 66’000-fold. The X-ray structure of Trx0P revealed a *trans* Ala76 bond and only small structural rearrangements relative to Trx1P, but the activity of Trx0P as electron transfer catalyst dropped about 100-fold relative to Trx WT. Our results show that optimal interaction with *in vivo* electron donors and acceptor substrates dominated over fast folding rates in the molecular evolution of thioredoxins.

## Results

### The X-ray structure of Trx0P shows how the Trx fold can adapt to a *trans* 75-76 peptide bond

**Figure 1:**
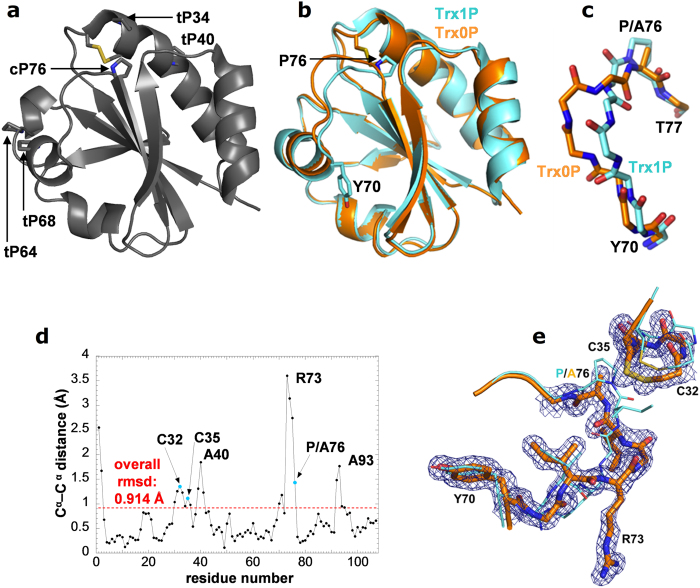
Comparison of the 1.65 Å X-ray structure of oxidized Trx0P (orange) with that of oxidized Trx1P (cyan; pdb ID 4HU7). **a**: Ribbon diagram of the X-ray structure of oxidized Trx WT. The side chains of the active-site cysteine pair (Cys32 and Cys35), the four *trans* prolines (tP34, 40, 64 and 68) and the single *cis* proline (cP76) are indicated in stick representation. **b**: Superposition of chain A of Trx0P with chain A of Trx1P, showing the conformational rearrangement in the loop segment 70–75 of Trx0P. The side chains of *cis* Pro76 and Tyr70 of Trx1P and the catalytic disulfide bond (Cys32–Cys35) of Trx0P are shown as stick representations. **c**: Comparison of the main chain segments 70–77 of Trx0P and Trx1P. The side chains of Pro76/Ala76 are also indicated. **d**: Plot of C_α_-C_α_ distances of the Trx0P/Trx1P pair against the residue number. **e**: Stick representations of the active-site segments C32–C35 and the segments Y70–A/P76 of Trx0P (thick orange lines) and Trx1P (thin cyan lines) in atom-specific colors. Segments C32–C35 and Y70–A76 of Trx0P are surrounded by 2mFo-DFc density, contoured at 1 σ.

To test the ability of the Trx fold of tolerating a *trans* peptide bond at the position of the conserved Pro76, the proline-free variant Trx0P was produced as soluble protein in *E. coli*, purified with high yields and crystallized. The X-ray structure of the oxidized form of Trx0P was solved at 1.65 Å resolution by molecular replacement using the structure of oxidized Trx1P (Trx1P_ox_) (PDB ID 4HU7)^18^ as search model. Data collection and refinement parameters are summarized in Supplementary Table 1. Overall, the structure of Trx0P_ox_ is highly similar to that of Trx WT_ox_ and Trx1P_ox_ (RMSDs of 0.724–0.915 Supplementary Table 2, Supplementary Fig. 1a, Supplementary Fig. 2) and shows the classical Trx fold with all regular secondary structure elements preserved (Fig. 1b). However, in contrast to Trx WT and Trx1P, the 75-76 peptide bond in Trx0P (Ile-Ala) adopts the *trans* conformation.

Notably, the 180° rotation around the 75–76 peptide bond in Trx0P relative to Trx1P is compensated by a structural rearrangement of the hexapeptide segment 70–75 N-terminal to the *trans* Ala76, which is part of the irregular secondary structure preceding β-strand 4. The position of β-strand 4 (residues 77–82) directly C-terminal to residue 76 is identical in the structures of Trx1P and Trx0P and an essential element of the central β-sheet of the thioredoxin fold, providing a plausible explanation for the fact that the polypeptide main chain around residue 76 only changed its conformation in the segment N-terminal to residue 76 in the Trx0P structure (Fig. 1b–e). The *trans* conformation of the 75–76 bond in Trx0P also translates into a slight, but significant conformational change in the polypeptide segment 30–35 harboring the active-site disulfide (Cys32 and Cys35), and structural rearrangements in the segments 39–41 and 92–95 (Fig. 1d). The overall r.m.s. deviation in the main-chain atoms (C_α_) between Trx1P and Trx0P is 0.914 Å (Fig. 1c,d), while it reaches values above 2.5 Å for R73, G74, and I75 next to the Pro76Ala replacement. The movement of the loop segment 70–75 likely causes a loss of van der Waals contacts with the active site disulfide, as Ile75 is no longer packed against the segment harboring the active site as a consequence of its 180° side chain flip (Fig. 1d and Supplementary Fig. 1a). An analogous variant of the periplasmic, thioredoxin-like oxidoreductase DsbA (Supplementary Fig. 1b) in which the conserved *cis* Pro151 was replaced by Ala, also resulted in formation of a *trans* peptide bond (Val150-Ala151)^19^. In contrast to Trx0P, however, the structural rearrangements accommodating the new *trans* peptide bond were even smaller and limited to the residues 149–152 (Supplementary Fig. 1b). Together, the structures of Trx0P and DsbA (Pro151Ala) reveal a surprisingly high tolerance of the Trx fold for a *trans* peptide bond at the position of its conserved *cis* proline.

### The *trans* peptide bond 75-76 in Trx0P leads to a dramatic loss in enzymatic activity

To characterize the functional consequences of the *trans* 75–76 peptide bond in Trx0P, we first compared the thermodynamic stabilities of the oxidized and reduced forms of Trx0P, Trx1P, and Trx WT at pH 7.0, 25 °C and a ionic strength (I) of 20 mM with guanidinium chloride (GdmCl)-induced unfolding and refolding transitions. All transitions proved to be fully reversible and were evaluated according to the two-state model of protein folding^20^ (Fig. 3a, Table 1). While the oxidized form of Trx WT was 12.0 ± 0.3 kJ mol^−1^ more stable than the reduced form, with a shift in the transition midpoint by 0.74 M GdmCl to higher GdmCl concentration, both redox forms of Trx1P showed essentially the same stability and transition midpoints (Fig. 3a, Table 1). Notably, the situation was reversed for Trx0P relative to Trx WT, where the reduced form was 7.6 ± 0.4 kJ mol^−1^ more stable than the oxidized form, showing an increase in the transition midpoint by 0.79 M GdmCl relative to Trx0P_ox_. The more stable oxidized form is a hallmark of Trx WT, which shows a highly reducing redox potential of −270 mV^21^,^22^. The formal thermodynamic linkage of the stability difference between the oxidized and reduced form of thioredoxin-like disulfide oxidoreductases with their disulfide exchange equilibrium constants with thiol/disulfide substrates^23^,^24^,^25^,^26^ predicted that Trx1P is more oxidizing than Trx WT, and that Trx0P is more oxidizing than Trx1P. This was indeed confirmed when we measured the redox potentials of Trx0P and Trx1P under identical conditions (pH 7.0, 25 °C, I = 20 mM) by equilibrium titration with mixtures of oxidized and reduced glutathione (GSSG and GSH, respectively). We determined the redox potentials of Trx0P and Trx1P to be −229 mV and −240 mV, respectively (Fig. 3b).

**Figure 2:**
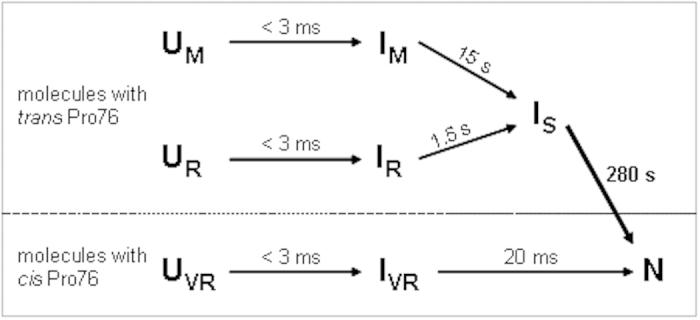
Proposed *in vitro* folding mechanism of *E. coli* thioredoxin, with half-lives of the individual reaction steps. (adapted from Georgescu *et al.* 1998)^16^.

**Figure 3:**
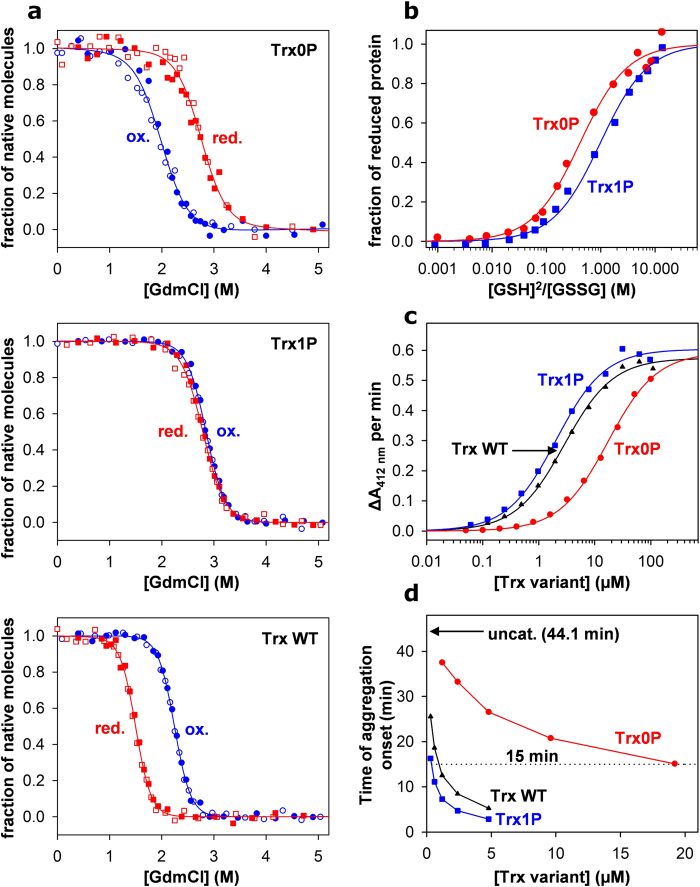
Stability and functional properties of Trx0P relative to Trx WT and Trx1P at 25 °C and pH 7.0. **a**: GdmCl-dependent equilibrium unfolding and refolding transitions (open and closed symbols, respectively) of the oxidized (blue circles) and reduced (red squares) proteins. Transitions were followed via the CD signal at 220 nm, fitted to the two-state model of folding (solid lines) and normalized. The deduced parameters D_1/2_ and m_eq_ with errors from the fits are given in Table 1. **b**: Redox potentials of Trx0P (red circles) and Trx1P (blue squares), determined via their equilibrium constants with GSH/GSSG. The fractions of reduced protein were obtained from fluorimetric data, plotted against the [GSH]^2^/[GSSG] ratio and fitted according to a disulfide exchange equilibrium (equation (3), solid lines), yielding equilibrium constants of 0.42 ± 0.05 M and 0.99 ± 0.11 M, and redox potentials (E^o^’) of −0.229 ± 2 mV and −0.240 ± 2 mV for Trx0P and Trx1P, respectively. The indicated errors result from fits according to equation (3). **c**: Kinetic analyses of Trx variants as substrates of thioredoxin reductase (TrxR). All variants show similar values of v_max_, but the K_M_ value for Trx0P (red circles) is increased 6.0 and 8.4-fold compared to that of TrxWT (black triangles) and Trx1P (blue squares), respectively (cf. Supplementary Table 3). The indicated errors are from fits according to the Michaelis Menten equation. **d**: The insulin reductase activity of Trx0P (red circles) is dramatically diminished relative to Trx1P and Trx WT (blue squares and black triangles, respectively). Kinetics of insulin aggregation as a result of Trx-catalyzed reduction by DTT were followed via the increase in optical density at 600 nm. The time of aggregation onset was plotted against catalyst concentration. Data were empirically fitted to a double exponential decay to identify the Trx concentration required to reduce the time of aggregation onset from 44.1 ± 0.3 min (uncatalyzed reaction) to 15 min (dashed line). The estimated error of the deduced Trx concentrations is 10% (see Supplementary Table 3).

Next, we compared the activities of Trx WT, Trx1P and Trx0P as substrates of thioredoxin reductase (TrxR) (Supplementary Table 3, Fig. 3c). All three Trx variants were reduced with very similar k_cat_ values (~10 s^−1^), but Trx0P showed a 6-fold and 8-fold increased K_M_ value compared to Trx WT and Trx1P, respectively. The small structural changes linked to the *trans* 75–76 bond thus made Trx0P a significantly worse substrate of TrxR. A similar result had been obtained when the *cis* Pro76 in Trx WT was replaced by Ala^17^. The results indicate that TrxR recognizes the WT-like tertiary structure of Trx with very high specificity. Trx0P also showed a dramatically reduced activity as catalyst of the reduction of insulin disulfides by dithiothreitol (DTT)^27^, which was decreased 17- and 55-fold relative to Trx WT and Trx1P, respectively (Fig. 2d, Supplementary Table 3, Supplementary Fig. 3a). This was clearly due to the slow reduction of insulin disulfides by reduced Trx0P, and not caused by slower regeneration of reduced Trx0P by DTT, as DTT reduced Trx0P even faster than Trx WT (Supplementary Table 3). The diminished reductase activity of Trx0P also agrees qualitatively with the more oxidizing redox potential of Trx0P relative to Trx WT and Trx1P. On the whole, despite the comparably small structural differences to Trx WT and Trx1P, the price to be paid for the *trans* 75–76 bond in Trx0P is strikingly high: the lower activities of Trx0P as acceptor of electrons from TrxR and, in particular, as donor of electrons to protein substrates predict that the *in vivo* activity of Trx0P would be about 100-fold lower compared to that of Trx WT.

### Trx0P folds four orders of magnitude faster than Trx1P

**Figure 4:**
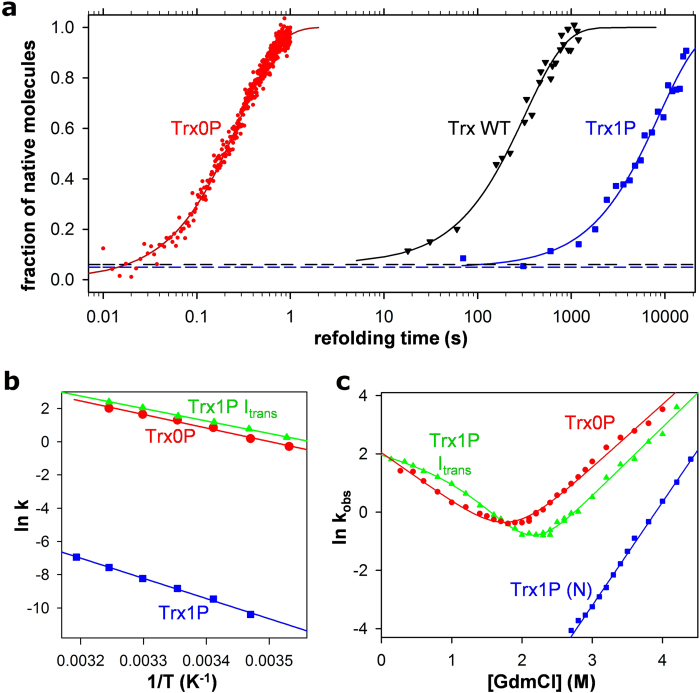
Folding kinetics of the oxidized forms of Trx WT, Trx0P, Trx1P and its *trans*-Pro76 intermediate (I_trans_) at 25 °C and pH 7.0. **a**: Kinetics of formation of N during refolding by dilution from 4.0 to 0.2 M GdmCl. Folding of Trx0P_ox_ (red dots) was consistent with a two-state mechanism (cf. panel C). A monoexponential fit (solid line) yielded an apparent rate constant of folding of 3.49 ± 0.01 s^−1^. Formation of N during refolding of Trx WT and Trx1P was recorded with interrupted refolding experiments (cf. Fig. S3). Trx WT (black triangles) showed 6% fast folding molecules (black dotted line), and 94% of the molecules reached N at a single rate of 3.10 ± 0.18 · 10^−3^ s^−1^ (solid line). Trx1P (blue squares) showed 5% fast folders (blue dotted line), and the residual 95% folded very slowly with a single rate of 1.16 ± 0.15 · 10^−4^ s^−1^. The indicated errors are standard errors from monoexponential fits. **b**: Arrhenius plot for folding of Trx0P (red circles), formation of I_trans_ from unfolded Trx1P (green triangles), and formation of native Trx1P from I_trans_ (blue squares), yielding activation energies of 67.6 ± 3.2, 62.4 ± 1.4 and 101.0 ± 2.8 kJ mol^−1^, respectively. The indicated errors are standard errors from Arrhenius fits. **c**: Chevron Plots showing the dependence of the apparent rate constant of unfolding/refolding (k_obs_) on [GdmCl] for Trx0P (red circles) and Trx1P I_trans_ (green triangles). Data were fitted according to a two-state model of protein folding in the case of Trx0P (solid, red line). Unfolding/refolding of I_trans_ was evaluated according to a three-state model^55^ with a high-energy on-pathway intermediate (solid, green line) (see legend to Table 1 for the deduced kinetic parameters). The unfolding branch of Trx1P is shown for comparison.

We next measured the folding kinetics for the oxidized forms of Trx WT, Trx1P and Trx0P at 25 °C and pH 7.0. For Trx0P unfolding and refolding was recorded by stopped-flow tryptophan fluorescence between 0.2 and 4 M GdmCl. Under all conditions, mono-exponential fluorescence traces without burst phases were obtained. Notably, a plot of the apparent rate constants of unfolding/refolding of Trx0P against GdmCl concentration (Fig. 4c) revealed a dynamic two-state folding equilibrium with a free energy of folding (ΔG^0^) of −17.4 ± 2.0 kJ mol^−1^ and a cooperativity (m_eq_) of 9.56 ±  0.40 kJ mol^−1^ M^−1^. The values are identical within experimental error to the corresponding parameters obtained with equilibrium unfolding of oxidized Trx0P (−18.8 ± 0.9 kJ mol^−1^ and 9.53 ± 0.43 kJ mol^−1^ M^−1^, respectively) (Fig. 3a, top panel; Fig. 4c, Table 1). The recorded fluorescence change during refolding thus directly reported the formation of native molecules, with an extrapolated rate constant of folding (k_F_) at zero denaturant of 7.70 ± 0.76 s^−1^, corresponding to half-life of 90 ms. Figure 4a shows the folding kinetics (normalized fluorescence trace) at 0.2 M GdmCl, with k_F_ = 3.5 s^−1^ and a half-life of 200 ms. The highly complex folding mechanism reported for Trx WT (Fig. 2) thus turned into the simplest possible mechanism through the replacement of all prolines by alanine.

Next, we monitored the kinetics of formation of N for Trx WT and Trx1P. Trx WT shows multiple kinetic phases during refolding in stopped-flow fluorescence experiments that however did not allow the quantification of native molecules^28^. We therefore applied interrupted refolding experiments that detect the concentration of N at any refolding time and the fractions of fast and slow refolding molecules^28^,^29^,^30^ (see Supplementary Fig. 3 for details). Trx WT and Trx1P were unfolded for at least 1.5 h at 25 °C and pH 7.0 in 4 M GdmCl to attain the *cis/trans* equilibrium of all prolyl peptide bonds before refolding was initiated by 1:20 dilution to 0.2 M GdmCl. The refolding was stopped after different times by addition of GdmCl to concentrations (2.88 M GdmCl for Trx WT, 3.45 M for Trx1P) where N unfolded with a half- life of about 30 s and any intermediate unfolded in the dead time of manual mixing. As both Trx WT and Trx1P refolded quantitatively under the chosen conditions, the amplitudes of the slow, monoexponential unfolding reactions could be converted to fraction of N and were plotted against refolding time. Figure 4a and Supplementary Fig. 3 show the kinetics of N formation for Trx WT and Trx1P compared to Trx0P at 0.2 M GdmCl. For Trx WT, the data were fully consistent with the previously proposed folding mechanism^15^ (Fig. 2) with a minor fraction of 6 ± 3% fast folders and a main fraction (94%) of slow folders. The slow folders accumulated a single, rate-limiting intermediate (I_S_ in Fig. 2) with Pro76 in *trans* that folded to N with a half-life of 222 ± 14 s, in good agreement with the previously reported half-life of 280 s under slightly different conditions^16^. Trx1P, for which we had expected a higher fraction of fast folders as it cannot accumulate wrong *cis* prolines at positions 34, 40, 64, and 68 in U, also only showed a fraction of 5 ± 2% fast folders. This is possibly due to residual structure affecting the *cis*/*trans* equilibrium of the Ile75-Pro76 peptide bond in the unfolded state. The major fraction of slow Trx1P folders reacted to N extremely slowly with a half-life of 99.1 ± 12.8 min. Thus, the four *trans* prolines in Trx WT accelerate the rate-limiting *cis*-to-*trans* isomerization of Pro76 27-fold relative to Trx1P. This may result from a significant change in the energy landscape through the replacement of the four *trans* prolines by *trans* alanines in Trx1P that even affects the late, rate-limiting folding reaction to the energetically most stable, native state^31^,^32^. More importantly, the results show that the single amino acid substitution Pro76Ala converting Trx1P into Trx0P accelerates folding to the thermodynamically most stable state (N) at zero denaturant by a factor of 66’000.

**Figure 5:**
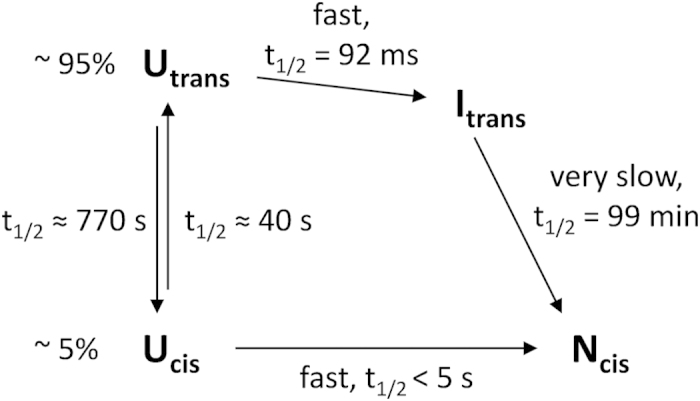
Minimal folding mechanism of Trx1P at pH 7.0 and 25 °C. Potential fast phases in the dead time of stopped flow mixing on the pathway from U_trans_ to I_trans_ and from U_cis_ to N_cis_ were not considered.

**Figure 6:**
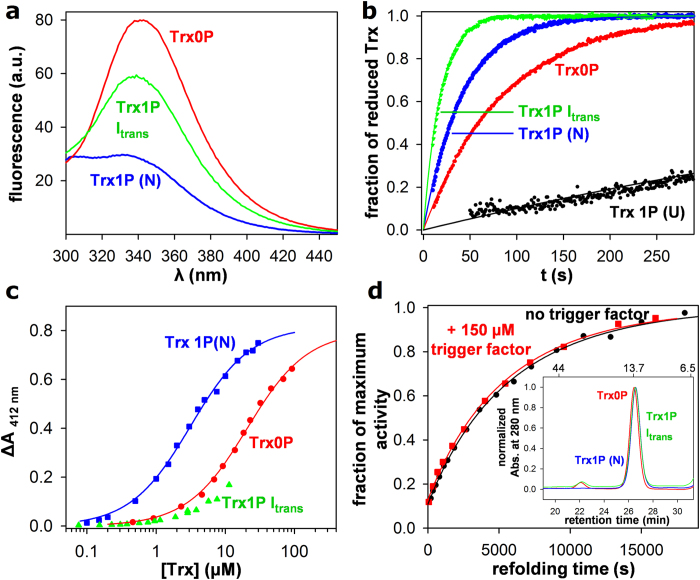
Biophysical and functional properties of Trx1P I_trans_ compared to Trx0P and native Trx1P at 0.2 M GdmCl, 25 °C and pH 7.0. **a**: Fluorescence spectra of Trx0P, native Trx1P and Trx1P I_trans_. **b**: Reduction of Trx0P, Trx1P, and Trx1P I_trans_ (2 μM each; red, blue and green dots, respectively) by 20 μM DTT, fitted according to second-order kinetics (solid lines) (see Supplementary Table 3 for the deduced rate constants and errors). Reduction of unfolded Trx1P with 100 μM DTT (black dots) revealed a rate constant of 10.3 ± 1.48 M^−1^ s^−1^) and was thus more than 100-fold slower than reduction of native Trx1P or I_trans_ (cf. Supplementary Table 3). **c**: Kinetic analysis documenting the properties of Trx0P (red circles), Trx1P (blue squares) and Trx1P I_trans_ (green triangles) as substrate of TrxR in the presence of 20 mM GdmCl at pH 8.0. Similar to Trx0P, I_trans_ showed an about 10-fold increase in K_M_ relative to that of Trx1P, but its catalytic parameters could not be determined accurately due to unspecific aggregation at higher concentrations (cf. Supplementary Table 3 for the parameters and errors deduced from Michaelis Menten fits). **d**: I_trans_ has a compact tertiary structure with a buried *trans* Ile75-Pro76 peptide bond that is inaccessible to catalysis by the PPIase trigger factor. Formation of native Trx1P from I_trans_ (90 μM) in the absence (black circles) and presence (red squares) of excess trigger factor (150 μM) was monitored by the increase in TrxR substrate activity and fitted monoexponentially (solid lines). Inset: Size exclusion chromatography on Superdex 75 of I_trans_, native Trx1P and native Trx0P (oxidized forms), showing that I_trans_ has same hydrodynamic volume as native Trx1P. The retention time of all proteins at ~26.5 min is about ¼ of the half-life of I_trans_ folding (99 min), guaranteeing that only a minor fraction of I_trans_ reacted to N during the chromatography. Retention times of molecular mass standard proteins (in kDa) are indicated on the top.

The folding kinetics of Trx1P at pH 7.0 and 25 °C are consistent with a simple folding mechanism in which U represents a mixture of 5% fast folders with Pro76 in *cis* (U_cis_) that fold directly to N, and 95% slow folders with Pro76 in *trans* (U_trans_) that collapse to a stable intermediate, I_trans_, with Pro76 still in *trans* (Fig. 5). We could reproduce the rate-limiting I_trans_-to-N reaction by two independent experiments within experimental error: the recovery of the activity of Trx1P as substrate of TrxR proceeded with a half-life of 79.5 ± 0.5 min, and the recovery of the tryptophan fluorescence of N (Fig. 6a) showed a half-life of 78.2 ± 0.5 min (Supplementary Fig. 4).

In addition, we used the strong fluorescence difference between I_trans_ and N (Fig. 6a) to measure the activation energy of the I_trans_-to-N reaction (Fig. 4b), which proved to be 101.0 ± 2.8 kJ mol^−1^. This compares to activation energies of 67.6 ± 3.2 kJ mol^−1^ for folding of Trx0P, and 62.4 ± 1.4 kJ mol^−1^ for the U_trans_-to-I_trans_ reaction (Fig. 4b).

To verify that folding of I_trans_ to N is indeed accompanied by the *trans*-to-*cis* isomerization of Pro76, we performed interrupted unfolding experiments in which Trx1P was rapidly unfolded by 5 M GdmCl (unfolding completed within 1 s) and incubated in 5 M GdmCl for different times (5–120 s) at 25 °C prior to refolding. The kinetics of attainment of the *cis*/*trans* equilibrium of the Ile75-Pro76 bond in unfolded Trx1P were measured via the increase in the fraction of slow folders with increasing incubation time (Supplementary Fig. 5) and yielded an apparent rate constant (k_obs_) of 1.80 ± 0.35 · 10^−2^ s^−1^, consistent with reported rates of *cis*-to-*trans* isomerization of prolyl peptide bonds in unstructured peptides^3^. Combination with the known fractions of fast and slow folders yielded half-lifes of 40 ± 7.7 s and 768 ± 382 s for the *cis*-to-*trans* and *trans*-to-*cis* isomerization of the Ile75-Pro76 peptide bond in unfolded Trx1P, respectively (Supplementary Fig. 5, Fig. 5).

### The intermediate I_trans_ has a highly stable tertiary structure

**Figure 7:**
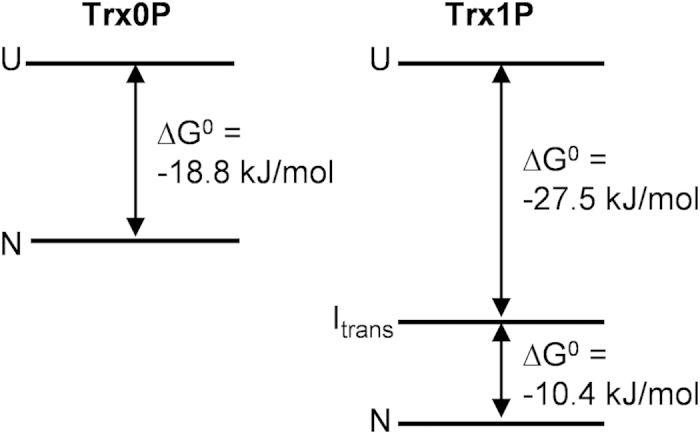
Energy diagrams of folding of Trx0P and Trx1P.

The rapid formation of I_trans_ during Trx1P refolding within the dead time of manual mixing, its high population (95% of the molecules) and in particular its extremely slow reaction to N offered the unique possibility to characterize the folding, stability and function of I_trans_ in detail. First, we recorded the GdmCl dependence of its unfolding and refolding kinetics by stopped-flow fluorescence (Fig. 4c). The fluorescence traces monitoring the formation of I_trans_ from unfolded Trx1P could be fitted mono-exponentially with an additional term correcting for the very slow formation of N from I_trans_ and showed no burst phase. Assuming that fluorescence changes resulting from the small fraction of 5% fast folding molecules were negligible, we assigned the first-order rate constants (k_obs_) deduced from the fast, main fluorescence change to formation of I_trans_. For recording the unfolding kinetics of I_trans_, it was first populated to >90% by rapid dilution of Trx1P from 4.0 to 0.2 M GdmCl (<5 min refolding time), and then unfolded again by increasing the GdmCl concentration. As native Trx1P unfolded by more than one order of magnitude slower than I_trans_ in the range of 2–4 M GdmCl (Fig. 4c) and was populated to less than 10% under these conditions, its contribution to the fast fluorescence change during unfolding of I_trans_ was minimal, so that all unfolded traces could be fitted mono-exponentially. Figure 4c shows the combined data on the GdmCl dependence of the apparent rate constant (k_obs_) of folding/unfolding of I_trans_, revealing a clear curvature of the folding branch of I_trans_. Evaluation according to a mechanism with a high-energy on-pathway folding intermediate^33^ yielded folding and unfolding rates of 7.52 ± 0.77 s^−1^ and 1.14 ± 0.62 · 10^−4^ s^−1^ at zero denaturant concentration, respectively and a free energy of folding (ΔG^0^) of −27.5 ± 2.4 kJ mol^−1^. In addition, the cooperativity of I_trans_ folding (m_kin_) of 12.7 ± 1.89 kJ mol^−1^ M^−1^ was identical within experimental error to the m_eq_ value of 13.3 ± 0.29 for Trx1P measured by equilibrium unfolding (Table 1), demonstrating complete tertiary structure formation in I_trans_. Together, I_trans_ proved to be even 8.7 kJ mol^−1^ more stable than Trx0P, and only 10.4 kJ mol^−1^ less stable than N (Table 1, Fig. 7). The tertiary structure of I_trans_ is likely very similar to that of native Trx0P, which also has the 75–76 peptide bond in *trans*. Comparison of the extrapolated rate constants of unfolding and refolding (Table 1) shows that the higher stability of Trx0P compared to I_trans_ is exclusively based on its 120-fold lower rate of spontaneous unfolding, as the proline-independent folding rates are essentially identical for I_trans_ and Trx0P (Fig. 4c, Table 1). We obtained further, independent evidence for a Trx0P-like tertiary structure of I_trans_. First, the tryptophan fluorescence spectrum of I_trans_ is more similar to that of Trx0P than to the spectrum of native Trx1P, which shows more than 2-fold lower fluorescence intensity than Trx0P (Fig. 6a). Second, the active-site disulfide bond in I_trans_ showed even a slightly higher reactivity with the reductant dithiothreitol (DTT) than that of native Trx1P and Trx0P, but a more than 200-fold higher reactivity with DTT than the disulfide bond in unfolded, oxidized Trx1P (Fig. 6b, Supplementary Table 3). Third, like Trx0P, I_trans_ was only weakly recognized as substrate by TrxR (Fig. 6c). Even though the catalytic parameters of I_trans_ could not be accurately determined due to aggregation at high concentrations during refolding, its K_M_ is similar to that of Trx0P (above 15 μM) and clearly increased relative to the K_M_ values of native Trx1P and Trx WT (Supplementary Table 3). Fourth, we performed fast analytical gel filtration runs showing that the apparent hydrodynamic volume of I_trans_ was indistinguishable from that of Trx1P and Trx0P within experimental error (Fig. 6d, inset). Together with the notion that folding becomes slower with increasing stability of structured intermediates^29^, the results suggested that the stable tertiary structure of I_trans_ is responsible for its very slow reaction to N.

To test whether I_trans_ can fold directly to N or first needs to unfold before the *trans*-to-*cis* isomerization of Pro76 and folding to N can take place, we measured the dependence of the kinetics of folding of I_trans_ to N on denaturant concentration. Supplementary Fig. 6 shows that N formed independently of GdmCl concentration between 0.2 and 1.0 M, demonstrating that unfolding of I_trans_ is not required for formation of N. In addition, we recorded refolding kinetics of Trx1P in the presence of a 1.67-fold molar excess of the ribosome-associated proline isomerase trigger factor (TF) from *E. coli.* TF is a likely catalyst of *cis/trans* isomerization of prolines during folding of thioredoxin *in vivo*, as it encapsulates nascent polypeptide chains leaving the ribosomal exit tunnel in a cavity that is large enough to accommodate the 12 kDa protein thioredoxin^34^,^35^. However, no acceleration of formation of native Trx1P by TF was observed, even at high concentrations of Trx1P (90 μM) and TF (150 μM) (Fig. 6d). This indicated that the *trans* Pro76 gets rapidly buried and inaccessible for TF upon the collapse of the polypeptide to I_trans_ and confirmed that N can be formed from I_trans_ directly. The inability of TF to catalyze folding of thioredoxin *in vitro* does however not question a catalytic function of TF *in vivo*, because ribosome-associated TF should be able to access prolines in disordered, nascent polypeptide chains that remain folding-incompetent prior to completion of protein synthesis.

## Discussion

Improved biological function is the critical driving force underlying molecular evolution of proteins^36^,^37^. Molecular evolution of the cytoplasmic reductase thioredoxin is a particularly interesting case because Trx not only needs to interact with its electron donor TrxR with very high efficiency (k_cat_/K_M_ > 10^6^ M^−1^ s^−1^, Supplementary Table 4), but also must donate electrons specifically to numerous substrates *in vivo*. These multiple functions define very stringent structural prerequisites for a functional Trx fold and, in particular, for the specific conformation and local environment of its active-site cysteine pair. In accordance with this notion, we showed that a single replacement of a buried amino acid (Pro76Ala) in the fully functional Trx variant Trx1P (yielding Trx0P) almost completely abolished the function of the protein as electron transfer catalyst. Although the re-orientation of the 75–76 bond from the *cis* to the *trans* conformation in Trx0P could be compensated by minor structural rearrangements and did not affect the overall protein fold, the small structural changes in the segment harboring the active-site disulfide were sufficient for an almost complete loss of function as TrxR-dependent reductase (Fig. 3, Supplementary Table 3). Inversely, the results indicate that the appearance of the *cis* Pro76 may have been a critical step in the evolution of Trx, in agreement with the finding that the diagnostic *cis* proline had already been present in the oldest bacterial thioredoxins^38^. But how could the *cis* proline evolve when it obviously affected the folding rate of Trx so dramatically? Our data on the rates of formation of N in the proline-independent folding of Trx0P and I_trans_, show that both proteins form N very rapidly with practically identical rates of ~8 s^−1^ (Fig. 4c, Table 1). Generation of a *cis* proline in the Trx fold should thus have slowed folding by at least two orders of magnitude. This may have been compensated by a native-like tertiary structure of I_trans_, proline isomerases as folding catalysts and molecular chaperones that increase the folding yield by preventing aggregation of slow folders. In addition, the cost of incorporation of a *cis* proline into a tertiary structure is formally comparably low (about 5 kJ mol^−1^), as the *trans* conformer of a prolyl peptide bond is energetically almost as unfavorable as the *cis* conformer^39^.

Figure 7 summarizes our results on the thermodynamics of Trx1P and Trx0P folding. The fact that Trx0P is less stable than Trx1P I_trans_ suggests a significant stabilization of the Trx fold by Pro76 relative to Ala76, even if Pro76 is in the nonnative *trans*-conformation. The comparably high free energy of folding of Trx1P I_trans_ also explains the slow formation of N from I_trans_.

The folding mechanism of Trx1P proved to be strongly simplified relative to that of Trx WT^16^ because the accumulation of nonnative *cis* prolines in U at the four *trans* prolines of Trx WT was eliminated. A further simplification was observed upon removal of the last proline in Trx0P, which became a perfect two-state folder. Our results are reminiscent of the elimination of one of the two *cis* prolines in ribonuclease T1, which also dramatically simplified RNaseT1 folding^40^. In addition, the 27-fold faster reaction of I_trans_ to N in Trx WT compared to Trx1P suggests that the four *trans* prolines in Trx WT destabilize I_trans_ in Trx WT relative to I_trans_ in Trx1P. The extremely slow I_trans_ to N reaction in Trx1P at the level of the intact tertiary structure underlines the strong coupling of folding with proline *cis*/*trans* isomerization^4^.

We showed that the replacement Pro76Ala in Trx1P accelerated folding of the vast majority (95%) of the molecules by a factor of 66’000. This is, to our knowledge, the largest acceleration of protein folding by a single amino acid replacement reported so far. The high acceleration factor is also a consequence of the extremely slow I_trans_-to-N reaction in Trx1P folding (t_1/2_ = 99 min). Indeed there are only very few examples for comparably slow folding reactions in small, one-domain proteins^28^,^41^. The very high activation energy of Trx1P folding might even provide the basis for designing a Trx variant that folds under kinetic control and stops folding at the level of I_trans_. Folding of Trx1P at 4 °C already proved to be close to kinetic control and reached N only with a half-life of 8 hours (Fig. 4b). Thus, even a slight, further stabilization of I_trans_ by additional amino acid replacements might suffice to achieve complete kinetic control of folding at 4 °C. Such thioredoxin variants could e.g. be used for technical applications where reductase activity is switched on by a temperature jump.

## Methods

### Protein production and purification

Trx WT and Trx1P were produced and purified as described previously^42^. The plasmid for expression of Trx0P was constructed with the QuikChange Site-directed Mutagenesis Kit (Agilent Technologies) using the expression plasmid for Trx1P2 as template. Production of Trx0P in *E. coli* BL21(DE3) and enrichment with anion exchange chromatography on DE52 cellulose (Whatman) and gel filtration on Superdex 75 (GE Healthcare) under non-reducing conditions was performed exactly as described for Trx WT and Trx1P^18^. After the gel filtration step, the remaining impurities in the combined fractions containing Trx0P were removed by hydrophobic chromatography on Phenyl Sepharose 6 Fast Flow (GE Healthcare) in 20 mM MOPS-NaOH pH 7.0, using a gradient from 1.5 to zero M ammonium sulfate (20 column volumes). Fractions containing pure Trx0P were combined, dialyzed against 5 mM MOPS-NaOH pH 7.0, and stored at −20 °C. The final yield of pure Trx0P was 11 mg per liter of bacterial culture. Ellman’s assay^43^ showed that the active-site disulfide bond in Trx0P was formed quantitatively after purification in the absence of reductant, and the correct mass of the protein was verified by ESI mass spectrometry (calculated: 11543.2 Da, found: 11543.3 Da). The concentrations of Trx WT, Trx1P and Trx0P were determined via their specific absorbance at 280 nm (14430 M^−1^ cm^−1^ for native, oxidized proteins and 14060 M^−1^ cm^−1^ for unfolded, oxidized proteins^44^).

*E. coli* thioredoxin reductase was purified as described previously,^45^ and its concentration was determined via its the specific absorbance at 280 nm (49000 M^−1^cm^−1^ for the monomeric holo-enzyme).

The genetic sequence encoding *E. coli* trigger factor with N-terminal hexahistidine tag was cloned into pET11a via NdeI and BamHI and expressed in *E. coli* BL21DE3 at 20 °C for 12 h after induction with 0.5 mM IPTG. After cell lysis in 50 mM potassium phosphate pH 8.0, 300 mM NaCl, 2 mM β-mercaptoethanol, 20 mM imidazole, the protein was purified via Ni-NTA affinity chromatography in the same buffer (elution with 40 mM imidazole-HCl), followed by anion exchange chromatography on Resource Q (GE Healthcare) (10 mM potassium phosphate pH 7.5, elution with an NaCl gradient at ~180 mM NaCl) and a final gel filtration step on Superdex 200 (GE Healthcare) in 20 mM Tris-HCl pH 7.5, 100 mM NaCl, 1 mM EDTA. The concentration of trigger factor (yield: 12 mg per liter of bacterial culture) was determined via its specific absorbance at 280 nm (17420 M^−1^ cm^−1^), and its identity was confirmed by ESI mass spectrometry (expected mass: 49015.5 Da; found: 49016.0 Da).

### Crystallization and structure determination of Trx0P_ox_

Crystals of Trx0P_ox_ were grown via sitting drop vapor diffusion at 4 °C after mixing 175 nl of Trx0P_ox_ solution (15.5 mg/ml in 20 mM MOPS-NaOH, pH 7.0) with 100 nl of precipitant solution (10% (w/v) PEG 1000, 10% (w/v) PEG 8000). Cryoprotection was achieved by shortly dipping the crystal into a solution of mother liquor with 20% ethylene glycol. Data to a resolution of 1.65 Å were collected at beamline X06DA of the Swiss Light Source (SLS, Villigen, Switzerland) at a wavelength of 1.0 Å. A complete dataset was obtained from a single crystal. Diffraction images were indexed and integrated with XDS^46^ in space group P2_1_ (a = 34.67 Å, b = 48.57 Å, c = 89.94 Å, α = β = 90°, γ = 101.01°), scaled with XSCALE and converted to amplitudes with XDSCONV and programs of the CCP4 suite^47^.

The three molecules of Trx0P in the asymmetric unit were positioned by molecular replacement with PHASER^48^, using the crystal structure of Trx1P_ox_ (PDB 4HU7)2 as search model. Refinement was carried out using PHENIX^49^. After the first round of refinement, clear difference electron density at the mutation site of P76A mandated the replacement of the *cis*-proline of Trx1P by a *trans*-alanine. MolProbity^50^ was used for structure validation; secondary structure elements were assigned using phenix.ksdssp. Diffraction data and refinement parameters are summarized in Supplementary Table 1.

### Denaturant-induced unfolding and refolding equilibria of Trx0P, Trx1P, and Trx WT

Unfolding and refolding equilibrium transitions were measured at 25 °C essentially as described previously^18^ with the only exception that 50 mM MOPS-NaOH pH 7.0, 1 mM EDTA was used as buffer. Protein concentrations were 16.6 μM. Samples with reduced proteins additionally contained 4 mM DTT. Exact GdmCl concentrations were determined via the refractive index of the solutions. Data were evaluated according to the two-state model of protein folding to determine the free energy (ΔG^0^) and cooperativity (m_eq_) of folding^20^.

### Interrupted refolding of Trx1P_ox_ and Trx WT_ox_

Interrupted refolding experiments (N-tests) were used to monitor formation of native molecules during refolding of Trx WT_ox_ or Trx1P_ox_, as they detect spectroscopically silent folding steps and quantitatively monitor the fraction of N at any time during refolding^30^. Proteins were first unfolded in 4.0 M GdmCl, 50 mM MOPS-NaOH pH 7.0, 1 mM EDTA at 25 °C for at least 2 h before initiation of refolding by rapid, 20-fold dilution with 50 mM MOPS-NaOH pH 7.0, 1 mM EDTA at 25 °C. The final concentrations in the refolding reactions were 35 μM Trx and 0.2 M GdmCl. Samples were removed after different refolding times and mixed with an equal volume of GdmCl solution in 50 mM MOPS-NaOH pH 7.0, 1.0 mM EDTA. The final GdmCl concentrations during this unfolding step were 2.88 M and 3.45 M for Trx WT_ox_ and Trx1P_ox_, respectively, and chosen such that native proteins unfolded with half-lives of about 30 s and folding intermediates (including I_trans_) unfolded within the dead time of manual mixing. Unfolding reactions at 25 °C were monitored via the increase in the far-UV CD signal at 220 nm with a J715 CD spectrometer (Jasco) and unfolding of N was fitted mono-exponentially (cf. Figure S4). The corresponding amplitudes were plotted against refolding time and normalized, yielding the kinetics of formation of N. The y-axis intercept yielded the fraction of fast folders (folding within the dead time of manual mixing). The folding kinetics of the slow folders could be fitted with a single exponential function for both TrxWT_ox_ and Trx1P_ox_, and were interpreted as the rate-limiting I_trans_-to-N transition.

### Unfolding and refolding kinetics of Trx0P_ox_ and Trx1P_ox_ I_trans_

The rates of unfolding and refolding of oxidized Trx0P and Trx1P I_trans_ were measured as a function of GdmCl concentration at 25 °C and pH 7.0 using a temperature-controlled SX20 stopped flow spectrophotometer (Applied Photophysics). The final protein concentration was 1.0 μM in all reactions. For refolding reactions, i.e., folding to N in the case of Trx0P and folding to I_trans_ in the case of Trx1P_ox_, proteins were first unfolded in 3.0 M GdmCl (Trx0P_ox_) or 3.6 M GdmCl (Trx1P_ox_) for at least 2 h at 25 °C, pH 7.0 and then refolded by 1:11 dilution with 50 mM MOPS pH 7.0, 1 mM EDTA containing different GdmCl concentrations. Refolding was recorded by the decrease in fluorescence above 320 nm (excitation at 280 nm). In the case of Trx0P_ox_, refolding rates were fitted according to a single, mono-exponential equation. In the case of Trx1P_ox_ I_trans_, a mono-exponential equation with an additional, linear term correcting for the slow fluorescence change caused by the subsequent slow proline-*trans*-to-*cis* isomerization was used. Unfolding of Trx0P_ox_ was performed by rapid mixing of the native protein with the 10-fold volume of 50 mM MOPS-NaOH pH 7.0, 1 mM EDTA containing different GdmCl concentrations, so that the final GdmCl concentrations varied between 2.1 and 4.0 M. Kinetics were fitted mono-exponentially. For recording unfolding kinetics of Trx1P_ox_ I_trans_, unfolded Trx1P_ox_ (in 4.0 M GdmCl) was refolded by dilution with 20 volumes of 50 mM MOPS-NaOH pH 7.0, 1 mM EDTA, and immediately unfolded again by 1:11 dilution into unfolding buffers resulting in final GdmCl concentrations of 2.3–4.2 M. Kinetic traces could be fitted mono-exponentially. The contribution of native Trx1P_ox_ (5% of the molecules) proved to be negligible. The logarithms of the apparent rate constants of folding and unfolding were plotted against GdmCl concentrations. Data on Trx0P were fitted according to the two state model of protein folding^51^. For Trx1P_ox_ I_trans_, data were fitted according to a model assuming a high-energy on-pathway intermediate (see legend to Table 1 for the deduced kinetic parameters)^33^.

### Fluorescence and CD spectroscopy

Fluorescence emission spectra of oxidized Trx0P, Trx1P, and Trx1P I_trans_ immediately after start of refolding (rapid dilution from 4.0 M GdmCl to 0.2 M GdmCl) were recorded at 25 °C in 50 mM MOPS-NaOH pH 7.0, 1 mM EDTA containing 0.2 M GdmCl using a temperature-controlled Quantum Master 7 fluorescence spectrometer (PTI). The excitation wavelength was 280 nm in all measurements.

Far-UV and near-UV CD spectra of oxidized Trx WT, Trx0P, and Trx1P were recorded in 5 mM KH_2_PO_4_-KOH pH 7.0 at 25 °C using a temperature-controlled J715 CD spectrometer (Jasco). The protein concentrations in far-UV and near-UV CD measurements were 0.2 mg/ml and 0.4 mg/ml, respectively. CD signals were converted to mean residue ellipticity. The far-UV CD spectrum of Trx1P I_trans_ was recorded immediately after the initiation of refolding of Trx1P at 25 °C in 50 mM MOPS-NaOH, 1 mM EDTA, 0.2 M GdmCl pH 7.0.

### Activity of Trx0P, Trx1P, and Trx1P I_trans_ as a substrate of thioredoxin reductase (TrxR)

The activities of Trx0P, Trx1P and Trx1P I_trans_ as substrates of TrxR at pH 8.0 and 25 °C were measured as described previously^45^ at constant TrxR concentrations of 40 nM (monomer), with the exception that 1 mM DTNB (5,5’-dithiobis-(2-nitrobenzoic acid), Ellman’s reagent) and 500 μM NADPH in a final reaction volume of 360 μl were used and all reactions contained 20 mM GdmCl. Samples of Trx1P_ox_ I_trans_ were obtained immediately after refolding of Trx1P_ox_ in 0.2 M GdmCl, 50 mM MOPS-NaOH pH 7.0, 1 mM EDTA). The reactions were initiated by addition of TrxR and followed by the absorbance increase at 412 nm as a result of the reduction of DTNB by reduced thioredoxin. The initial velocities were plotted against substrate concentrations and evaluated according to Michaelis-Menten kinetics. For calculation of k_cat_ values, a molar extinction coefficient of 28300 M^−1^cm^−1^ was used for the two TNB molecules formed per catalytic cycle.

As an alternative to the N-test, the rate of formation of native Trx1P_ox_ from I_trans_ was monitored by measuring the increase in the activity as substrate of TrxR as a function of refolding time. Refolding of unfolded Trx1P_ox_ was started as described above (10 μM Trx in the refolding reaction containing 0.2 M GdmCl) at 25 °C and pH 7.0. After different refolding times, samples were removed and diluted 1:10 with TrxR reaction buffer (0.1 M Tris-HCl pH 8.0, 0.25 mM EDTA, 0.25 mg/ml BSA, 1 mM DTNB, 0.5 mM NADPH), resulting in final concentrations of 1 μM Trx and 20 mM GdmCl in the TrxR activity assay. The initial reaction velocities were plotted against refolding time and the rate of formation of native Trx1Pox was determined assuming Michaelis-Menten type substrate activity for I_trans_ and native Trx1P according to equation (1),




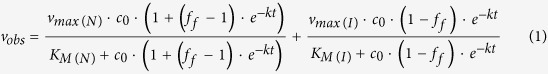




where v_obs_ is the observed initial reaction velocity, v_max(N)_ and v_max(I)_ are the maximum reaction velocities of native Trx1P and Trx1P I_trans_, respectively, c_0_ is the total concentration of Trx1P, f_f_ is the fraction of fast folding molecules (0.05), k is the rate constant of the I_trans_-to-N reaction, and K_M(N)_ and K_M(I)_ are the Michaelis constants of native Trx1P and Trx1P I_trans_, respectively. v_max(I)_ was assumed to be identical to v_max(N)_ based on the observation that v_max_ with Trx0P as substrate proved to be identical to v_max(N)_ of native Trx1P.

### Rate of attainment of of the *cis/trans* equilibrium of Pro76 in unfolded Trx1P

The rate of proline *cis*-to-*trans* isomerization in unfolded proteins can be determined by interrupted unfolding experiments^30^,^52^. First, native Trx1P_ox_ (100% *cis* Pro76) was rapidly unfolded at 25 °C and pH 7.0 in 5.0 M GdmCl (unfolding half-life < 1 s). After different incubation times in 5 M GdmCl, refolding was initiated by rapid dilution with 50 mM MOPS-NaOH, 1 mM EDTA pH 7.0 to a final GdmCl concentration of 0.2 M). TrxR substrate activity of Trx1P_ox_ during refolding was recorded as described above and the activity increase as a function of refolding time was fitted mono-exponentially. As a consequence of formation of *trans* Pro76 in unfolded Trx1P_ox_, the amplitudes of the slow activity increase of Trx1P_ox_ as TrxR substrate during refolding increased with unfolding time (cf. Figure S5A). The amplitudes were plotted against unfolding time (Supplementary Fig. 5B) and yielded the apparent rate constant (k_obs_) of attainment of the *cis/trans* equilibrium of Pro76 in unfolded Trx1P_ox_.

### Kinetics of reduction of Trx WT and variants by DTT

Kinetics of reduction of oxidized Trx0P, Trx1P, or Trx1P I_trans_ by DTT were recorded at 25 °C in 50 mM MOPS-NaOH, pH 7.0 under pseudo-first-order conditions. Oxidized thioredoxins (2.0 μM) were mixed with 200, 100, 50 or 20 μM DTT in a temperature-controlled Quantum Master 7 fluorescence spectrometer. For each Trx variant, the increase in tryptophan fluorescence at 345 nm (excitation at 280 nm) upon reduction was fitted globally to pseudo-first-order kinetics. The kinetics of reduction of unfolded Trx1P could also be followed by an increase in tryptophan fluorescence, which is most likely caused by quenching of the fluorescence of Trp31 by the Cys32-Cys35 disulfide in the unfolded protein.

### Redox equilibria with glutathione

The redox equilibrium between Trx0P or Trx1P and GSH/GSSG (eq. 2) was determined essentially as described previously^42^, making use of the increase in tryptophan fluorescence upon reduction of Trx and assuming that mixed disulfides between Trx and glutathione are not significantly populated at equilibrium. Trx1P_ox_ or Trx0P_ox_ (2 μM) were mixed with GSSG (10 μM) and variable concentrations of GSH (0.015–33 mM). After incubation under argon atmosphere for 5 h at 25 °C in 100 mM KH_2_PO_4_/K_2_HPO_4_ pH 7.0, 1 mM EDTA, the fluorescence at 345 nm was recorded (excitation at 295 nm). As the GSH preparation contained small impurities of GSSG, the true GSSG concentration in all samples was determined immediately after each fluorescence measurement via glutathione reductase-catalyzed NADPH oxidation as described previously^53^. The equilibrium constant (K_eq_, eq. 2) was determined by fitting the fluorescence data according to equation 3, where F is the measured fluorescence and F_ox_ and F_red_ are the fluorescence intensities of oxidized and reduced Trx, respectively. Redox potentials were calculated with the Nernst equation (eq. 4), using a value of −240 mV for the standard redox potential of glutathione^54^.




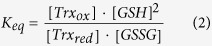







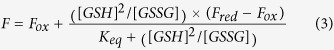







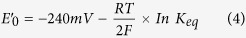




### Thioredoxin-catalyzed reduction of insulin by DTT

The activity of Trx0P and Trx1P as catalysts of the reduction of horse insulin (130 μM) by DTT (1 mM) was measured at 25 °C and pH 7.0 as described previously^18^, except that a final DTT concentration of 1.0 mM was used. The final concentration of Trx was varied between zero and 20 μM. The aggregation of insulin as a consequence of reduction of insulin disulfides was followed via the increase in optical density (OD) at 600 nm. The time required to reach an OD_600_ nm value of 0.1 was defined as the time of aggregation onset and plotted against Trx concentration. The time of aggregation onset was 44.1 ± 0.3 min for the uncatalyzed reaction.

### Data analysis and statistics

The quantitative evaluation of the data provided in Table 1 and Figs 3,4 and 6 was performed according to the equations/models indicated in the figure legends, using Kaleidagraph (Synergy Software) or Sigmaplot (Systat Software Inc.). Evaluation of the folding kinetics of I_trans_ according to a high-energy on-pathway intermediate was performed with proFit (QuantumSoft). If not stated otherwise, the given errors result from the fits to the indicated equations/models.

## Additional Information

**How to cite this article**: Roderer, D. J. A. *et al.* Acceleration of protein folding by four orders of magnitude through a single amino acid substitution. *Sci. Rep.* **5**, 11840; doi: 10.1038/srep11840 (2015).

## Supplementary Material

Supplementary Information

## Figures and Tables

**Table 1:** Thermodynamic and kinetic parameters of folding of thioredoxin variants 25 °C and pH 7.0.

Thermodynamic parameters of GdmCl-dependent unfolding/refolding equilibria of the thioredoxin variants, and folding rates Trx WT_ox_ and Trx1P_ox_ involving the *trans*-to-*cis* isomerization of Pro76*												
Trx variant			m_eq_ (kJ mol^−1^ M^−1^)		D_1/2_ (M GdmCl)		ΔD_1/2_^a^ (M GdmCl)		ΔΔG^0^_ox/red_ at the mean D_1/2_^b^ (kJ mol^−1^)		k_Itrans→Ncis_ (s^−1^)^c^	
Trx WT_ox_			15.9 ± 0.34		2.24		0.74		12.0 ± 0.3		3.11 ± 0.20 · 10^−3^	
Trx WT_red_			16.5 ± 0.50		1.50						n.d.^g^	
Trx0P_ox_			9.53 ± 0.43		1.97		−0.79		−7.6 ± 0.4		n.a.^h^	
Trx0P_red_			9.82 ± 0.64		2.76						n.a.^h^	
Trx1P_ox_			13.3 ± 0.29		2.85		0.06		0.8 ± 0.1		1.16 ± 0.15 · 10^−4^	
Trx1P_red_			11.8 ± 0.32		2.79						n.d.^g^	
**Kinetic parameters of unfolding and refolding that are independent of proline cis/trans isomerization** **												
**Trx variant**	**k**_**F**_^**H_2_O**^ **(s**^**−1**^)		**k**_**U**_^**H_2_O**^ **(s**^**−1**^)		**m**_**F**_^d^ **(M**^**−1**^)		**m**_**U**_ d **(M)** ^**−1**^		**ΔG**^**0**^_**kin**_ **(kJ mol**^**−1**^)		**m**_**kin**_ **(kJ mol**^**−1**^ **M**^**−1**^)	
Trx0P_ox_	7.70 ± 0.76		7.00 ± 1.3 ∙ 10^−3^		−1.69 ± 0.10		2.17 ± 0.06		−17.4 ± 2.0		9.56 ± 0.40^e^	
Trx1P_ox_	n.d.^g^		9.56 ± 0.12 ∙ 10^−7^		n.d.^g^		3.55 ± 0.05		n.d.^g^		n.d.^g^	
Trx1P_ox_ I_trans_	7.52 ± 0.77^f ^		1.14 ± 0.62 ∙ 10^−4^ f		n.a.^h^		n.a.^h^		−27.5 ± 2.4^f^		12.7 ± 1.89^f^	

^a^Difference between the transition midpoints of both redox forms, defined as D_1/2 (ox)_−D_1/2 (red)_.

^b^To avoid errors due to extrapolation to zero denaturant, the differences between the free energies of folding of the oxidized and reduced forms was calculated at the GdmCl concentrations corresponding to the mean value of the respective transition midpoints (D_1/2 mean_). D_1/2 mean_ values were 1.87, 2.37 and 2.82 M GdmCl for Trx WT, Trx0P and Trx1P, respectively. The ΔΔG^0^_ox/red values_ are defined such that positive values mean that the oxidized form is more stable than the reduced form.

^c^Rate constant of the rate-limiting step in the folding of the majority of molecules (94% in the case of Trx WT_ox_ and 95% in the case of Trx1P_ox_) involving the *trans*-to-*cis* isomerization of the Ile75-Pro76 peptide bond, determined in the presence of 0.2 M GdmCl by interrupted refolding experiments (cf. Fig. 4a and Supplementary Fig. 3).

^d^Kinetic m values, corresponding to the linear dependence of ln k_F_ and ln k_U_ on GdmCl concentration.

^e^Equilibrium m value predicted from kinetic data with the equation m_kin_ = (m_U_–m_F_)·RT.

^f^The GdmCl dependence of the observed rate of folding/unfolding of I_trans_ (Fig. 4c) was evaluated according to a mechanism with a high-energy on-pathway intermediate (U↔I↔N) with k_F_ = k_UI_ and k_U_ = k_NI_(k_IU_/k_IN_)^33^): m_kin_ = (Σ|m_i_|) · RT, with m_UI_ = −0.58 M^−1^, m_IN_ = −2.22 M^−1^, m_NI_ = 2.33 M^−1^ (m_IU_ was fixed to zero); k_UI_ = 7.52 s^−1^, k_IN_ = 1.44 · 10^5^ s^−1^, and k_NI_ = 1.64 · 10^−3^ s^−1^ (k_IU_ was fixed to 10^4^ s^−1^). The deduced transition midpoint of I_trans_ is 2.17 M GdmCl.

^g^not determined.

^h^not applicable.

^*^Indicated errors correspond to errors from the fits described in the legends of Figs 3a and 4a.

^**^Indicated errors correspond to errors from the fits decribed in the legends to Fig. 4a,c.
