# Supplementary material for: Acceleration of protein folding by four orders of magnitude through a single amino acid substitution
Source: Sci Rep. 2015 Jun 30;5:11840. doi: 10.1038/srep11840 (PMC4485320; doi:10.1038/srep11840)
Supplement: Supplementary Information [file srep11840-s1.pdf]

Acceleration of protein folding by four orders of magnitude through a single amino acid substitution

Daniel J. A. Roderer[1‡§], Martin A. Schärer[1‡†], Marina Rubini[2*] and Rudi Glockshuber[1]

AUTHOR ADDRESS

[1] ETH Zurich, Institute of Molecular Biology and Biophysics, Otto-Stern-Weg 5, CH-8093 Zurich, Switzerland

[2] University of Konstanz, Department of Organic Chemistry, Universitätsstrasse 10, D-78464 Konstanz, Germany

*marina.rubini@uni-konstanz.de

[‡] These authors contributed equally

Current address: [§] Max Planck Institute of Molecular Physiology, Department of Structural Biochemistry, Otto-Hahn-Strasse 11, D-44227 Dortmund; [†] Paul Scherrer Institut, CH-5232 Villigen, Switzerland

Supplementary Information

SUPPLEMENTARY TABLES

Supplementary Table 1: Statistics of the X-ray structure determination of oxidized Trx0P.

	**Trx0P: pdb ID 4X43**
Space group	P 1 21 1
Cell dimensions	
a, *b*, *c* (Å)	34.67, 48.57, 89.94
α, β, γ (°)	90.00, 101.01, 90.00
Resolution (Å)	50.0 – 1.65 (1.69 – 1.65) [i]
ISa	30.8
R_{meas} (%)	14.5 (212.0) [i]
$CC_{1/2}$	99.9 (40.0) [i]
$I / \sigma I$	14.0 (1.1) [i]
Completeness (%)	99.9 (99.6) [i]
Redundancy	12.3 (11.9) [i]
Wilson B ($Å^2$)	28.5
**Refinement**	
Resolution (Å)	44.1 – 1.65
No. reflections[ii]	35498 (924) [i]
R_{work} / R_{free}	17.6 / 21.8
No. atoms	
Protein	2464
Water	284
B-factors ($Å^2$)	
Protein	28.5
Water	34.3
Ramachandran analysis[iii]	
Favored (%)	98.4
Allowed (%)	1.6
R.m.s. deviations	
Bond lengths (Å)	0.008
Bond angles (°)	1.016

Data was collected from a single crystal.
[a]Values in parentheses are for the highest-resolution shell.
[b]Number of reflections in test set.
[c]Ramachandran analysis was performed with PHENIX.

Supplementary Table 2: RMS deviations (CA atoms, complete structures) between the three different Trx0P$_{ox}$ molecules in the asymmetric unit of the Trx0P$_{ox}$ structure (PDB ID 4X43) and the two molecules in the asymmetric unit of the Trx1P$_{ox}$ structure (4HU7), calculated with SSM superpose as implemented in COOT.

	Trx0P_B	Trx0P_C	Trx1P_A	Trx1P_B
**Trx0P_A**	0.575	0.399	0.744	0.751
**Trx0P_B**		0.482	0.915	0.804
**Trx0P_C**			0.772	0.724

Supplementary Table 3: Comparison between the kinetic parameters of the active-site reactivity (Cys32 and Cys35) of Trx WT, Trx1P, Trx1P I_{trans} and Trx0P.

Trx variant	Trx WT	Trx1P	Trx1P I_{trans}	Trx0P
Activity as substrate of thioredoxin reductase at 25 °C, pH 8.0 and zero GdmCl (cf. Figure 3C)				
k_{cat} (s^{-1})	10.2 ± 0.12	10.8 ± 0.16	n.a.	10.6 ± 0.18
K_M (µM)	2.86 ± 0.15	2.04 ± 0.14		17.2 ± 0.85
k_{cat}/K_M ($M^{-1}s^{-1}$)	$2.95 \cdot 10^6$	$4.35 \cdot 10^6$	n.a.	$5.08 \cdot 10^5$
reactivity with TrxR relative to Trx WT	1.0	1.5	n.a.	0.17
Activity as substrate of thioredoxin reductase at 25 °C, pH 8.0 and 0.02 M GdmCl (cf. Fig. 6c) [a]				
k_{cat} (s^{-1})	n.d.	12.1 ± 0.29	n.d.	11.8 ± 0.30
K_M (µM)	n.d.	3.13 ± 1.48	≥ 20 [a,b]	20.7 ± 0.99
k_{cat}/K_M ($M^{-1}s^{-1}$)	n.d.	$3.86 \cdot 10^6$		$5.70 \cdot 10^5$
Reactivity of the active site-disulfide with DTT at pH 7.0 and 25 °C in 0.2 M GdmCl (cf. Fig. 6b) [b]				
k_{DTT} ($M^{-1}s^{-1}$)	178 ± 12	1173 ± 117	2432 ± 385	544 ± 44
reactivity with DTT relative to Trx WT	1	6.6	13.7	3.1
Reactivity with DTT relative to unfolded Trx1P [c]	n.a.	114	236	n.a.
Insulin reductase activity (cf. Fig. 3d)				
$c_{44min \rightarrow 15min}$ (µM) [d]	0.88 ± 0.10	0.36 ± 0.04	n.a.	19.7 ± 2.0
reductase activity relative to Trx WT	1	2.44	n.a.	0.045

[a] TrxR-assays were performed in 0.02 M GdmCl after accumulation of I_{trans} by 20-fold dilution of Trx1P from 4 M to 0.2 M GdmCl, followed by another 10-fold dilution with the TrxR assay buffer (final GdmCl concentration: 20 mM). Due to aggregation of I_{trans} at concentrations above 150 µM in 0.2 M GdmCl and the strong dependence of TrxR activity on GdmCl, its K_M could not be accurately determined. Assuming a wild-type like k_{cat} value, a value of 15 µM the can be indicated as lower limit for the K_M of I_{trans} (cf. Fig. 6c).

[b] Determined at 25 °C and pH 7.0 in the presence of 0.2 M GdmCl (cf. Fig. 6b).

[c] The disulfide bond of unfolded, oxidized Trx1P was reduced by DTT in 4 M GdmCl, and reacted with a rate constant of 10.3 ± 1.5 $M^{-1}s^{-1}$ at pH 7.0 and 25°C (cf. Fig. 6c).

[d] Catalyst concentration required to reduce the time of insulin aggregation onset from 44.1±0.3 min (uncatalyzed reaction; the error corresponds to the standard deviation of three independent measurements) to 15 min (cf. Fig. 3d).

n.a.: not applicable; n.d.: not determined.

SUPPLEMENTARY FIGURES

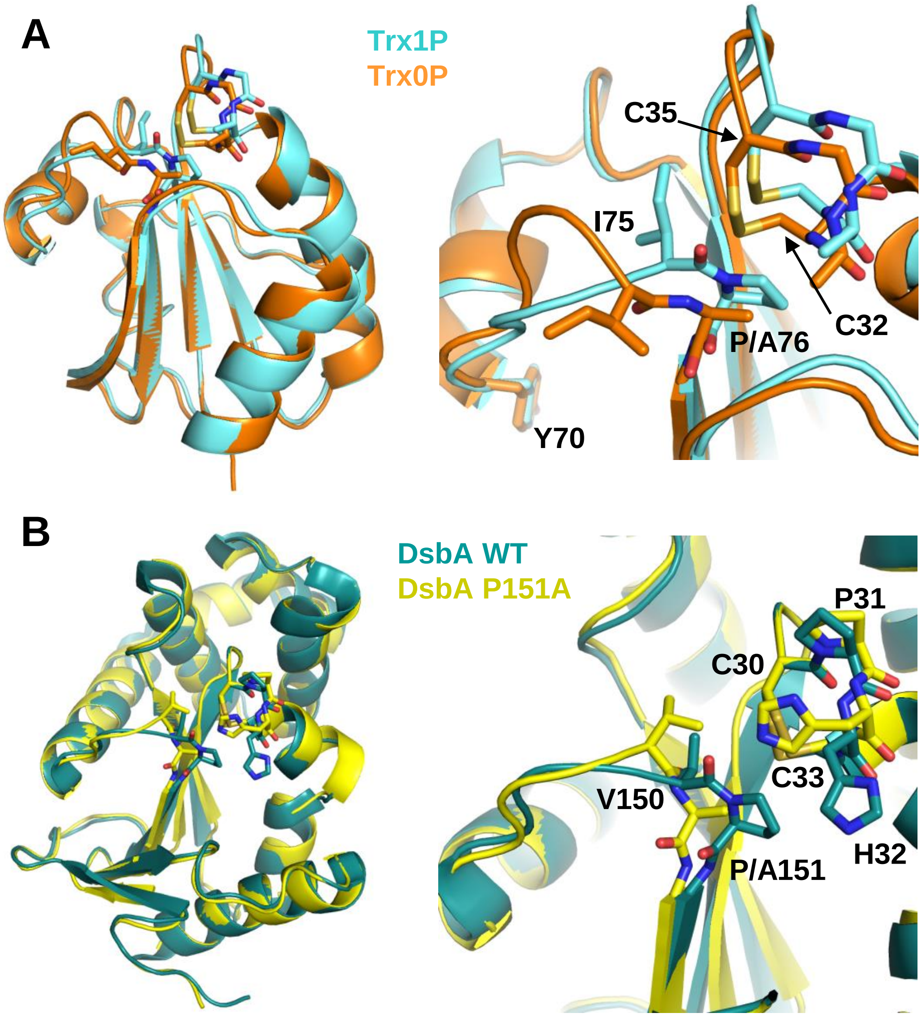

Supplementary Figure 1: Comparison of the structural rearrangements in *E. coli* thioredoxin (A) and *E. coli* DsbA (B) occurring upon replacement of the conserved *cis* proline (Pro76 and Pro151, respectively) in their Trx fold by alanine. A: Superposition of chain A of Trx0P$_{ox}$ (orange) with chain A of Trx1P$_{ox}$ (cyan; pdb ID 4HU7), showing that the main chain in the segment 70–76 changes its conformation in Trx0P as a consequence of the *trans* Ile75-Ala76 bond. The side chains of Tyr70, Ile75, Pro/Ala76 and the active-site disulfide bonds between Cys32 and Cys35 are shown as stick representations in both structures. Note the opposite side chain orientation of Ile75 as a result of the *trans* peptide bond 75-76 and the shift of the active-site disulfide bond. B: Superposition of the X-ray structure of oxidized wild type DsbA (pdb ID 1FVK, turquoise) with that of its P151A variant[18] (pdb ID 1BQ7, yellow). The side chains of Val150, Pro/Ala151 and the active sites (Cys30–Cys33) are shown as stick representations. The figure shows that the adaptation of DsbA to the new *trans* 150-151 peptide bond in its Pro151Ala variant is essentially restricted to conformational rearrangements in the short tetrapeptide segment 149–152.

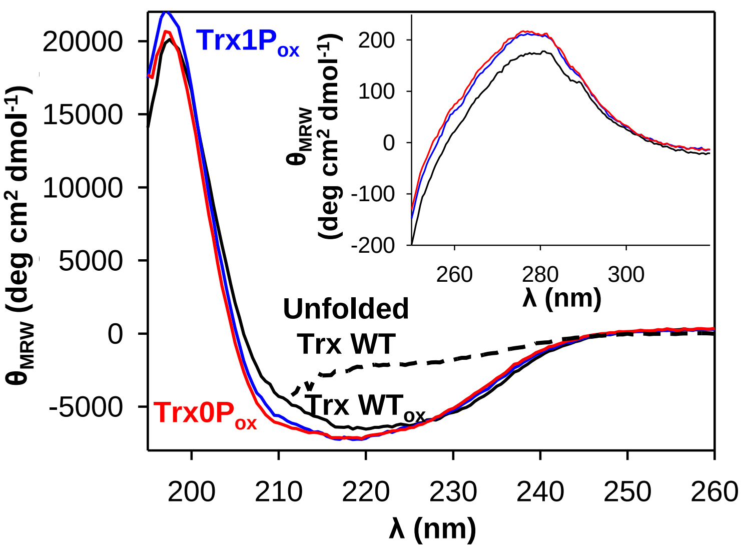

Supplementary Figure 2: Comparison of the far-UV and near-UV (inset) CD spectra of Trx0P$_{ox}$ (red) with those of Trx WT$_{ox}$ (black) and Trx1P$_{ox}$ (blue) at pH 7.0 and 25°C. Protein concentrations of 0.2 mg/ml and 0.4 mg/ml in 5 mM KH_2PO_4-KOH pH 7.0 were used for recording far- and near-UV CD spectra, respectively. In addition, the far-UV CD spectrum of unfolded Trx WT$_{ox}$ in 4.0 M GdmCl is shown for comparison.

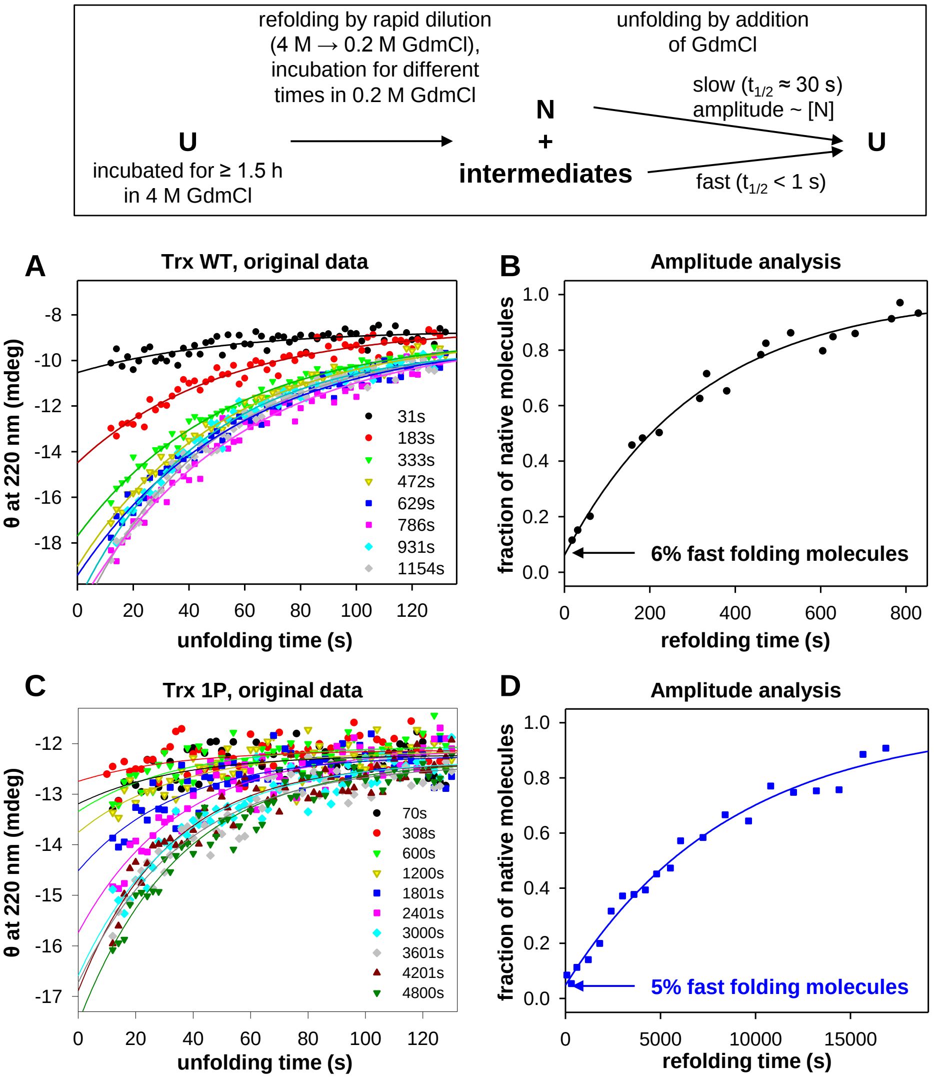

Supplementary Figure 3: **Kinetics of formation of native molecules during refolding of oxidized Trx WT (A, B) and oxidized Trx1P (C, D) at 25°C and pH 7.0, determined by interrupted refolding experiments (N-tests).** Top: Scheme describing the individual steps in the interrupted refolding experiments. A, C: Unfolding kinetics of Trx WT (A) and Trx1P (C) recorded after the indicated times of refolding, recorded via the increase in the CD signal at 220 nm (cf. Supplementary Figure 2). B, D: Fraction of native molecules of Trx WT (B) and Trx1P (D) plotted against refolding time (see also Fig. 4a). The amplitudes of the CD traces shown in A and C were plotted against refolding time, fitted mono-exponentially (solid lines) and then normalized. Extrapolation to zero refolding time reproducibly yielded 6±3% fast folders for Trx WT_{ox} (B) and 5±2% fast folders for $Trx1P_{ox}$ (D).Native proteins were first unfolded in 4.0 M GdmCl and incubated for ≥1.5 h in 4.0 M GdmCl to attain the *cis/trans* equilibria of the prolyl peptide bonds in the unfolded proteins. Refolding was initiated by rapid dilution with a 20-fold volume of 50 mM MOPS-NaOH pH 7.0, 1 mM EDTA (final GdmCl concentration: 0.2 M). After different times of refolding, proteins were unfolded again by 1:1 dilution with 50 mM MOPS-NaOH pH 7.0, 1 mM EDTA containing GdmCl such that final GdmCl concentrations of 2.88 M (Trx WT) and 3.45 M (Trx1P) were obtained. At these GdmCl concentrations, the native proteins (with *cis* Pro76) unfold

with half-lifes of about 30 s, while all folding intermediates unfolded within the dead time of manual mixing.

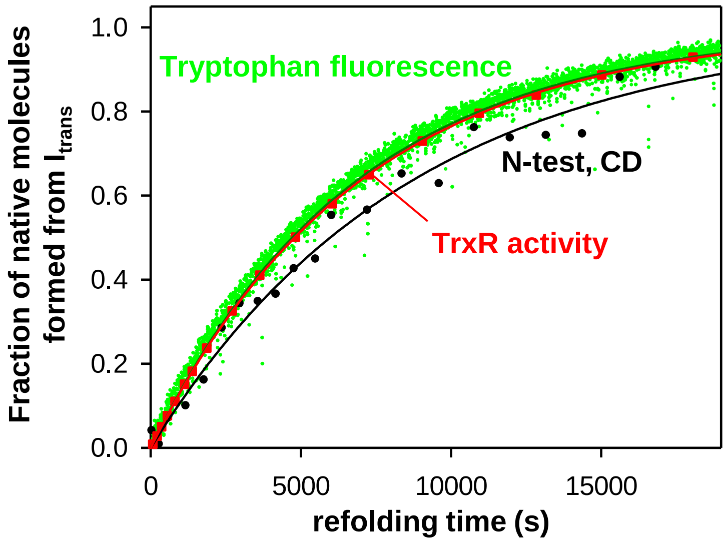

Supplementary Figure 4: **Kinetics of the rate-limiting step of Trx1P$_{ox}$ folding (I_{trans}→N_{cis}), measured by three independent methods and yielding the same rate constants within experimental error.** The black circles correspond to the kinetics of the I_{trans}→N_{cis} reaction measured by interrupted refolding (cf. Fig. 4a and Supplementary Fig. 3c,d). The red squares represent the I_{trans}→N_{cis} reaction measured via the increase in the activity as substrate of TrxR. The green dots represent the I_{trans}→N_{cis} transition monitored via the decrease in Trp fluorescence (cf. Fig. 6a). The half-lifes of the I_{trans}→N_{cis} transition, deduced from single-exponential fits (solid lines) were 99.1 ± 12.8 min, 79.5 ± 0.5 min and 78.2 ± 0.5 min for the N-test, the test for TrxR substrate activity and the Trp fluorescence kinetics, respectively.

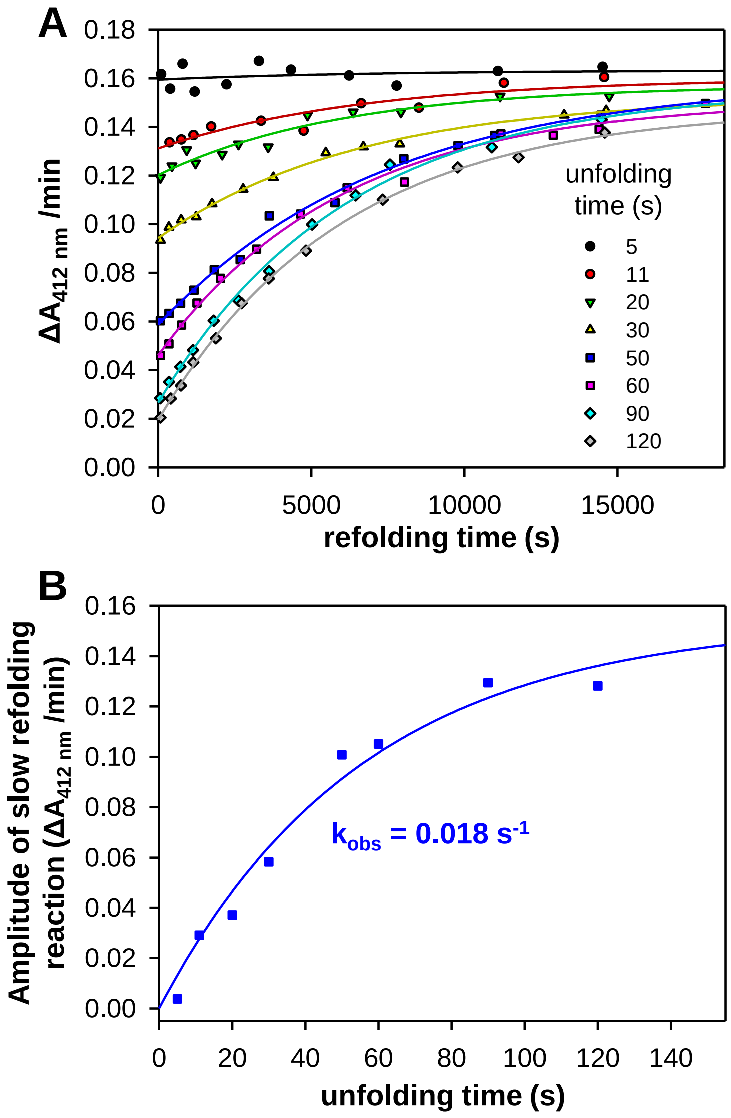

Supplementary Figure 5: **Kinetics of attainment of the *cis/trans* equilibrium at pH 7.0 and 25 °C of the Ile75-Pro76 peptide bond in unfolded Trx1P, recorded by interrupted unfolding.**

A: Native Trx1P (with 100% *cis* Pro76) was rapidly unfolded with 5 M GdmCl (unfolding was completed within 1 s). After the indicated incubation times in 5 M GdmCl, Trx1P was refolded by dilution to 0.2 M GdmCl, and the increase in its activity as substrate of thioredoxin reductase (TrxR) (given in ΔA_{412nm}/min) with refolding time was recorded. The kinetics were fitted with a model assuming a mixture of fast folders (with Pro76 in *cis*) that refold in the dead time of manual mixing, and slow folders (with Pro76 in *trans*) that fold to N in a mono-exponential reaction (cf. equation 1).

B: Plot of the amplitudes of the slow refolding reactions from panel A against unfolding time at 25 °C and pH 7.0 in 5.0 M GdmCl. The data were fitted according to first-order kinetics, yielding an apparent rate constant (k_{obs}) of $1.80 \pm 0.35 \cdot 10^{-2}$ s^{-1} for the attainment of equilibrium. The rate constant k_{obs} corresponds to the sum of the microscopic rate constants $k_{cis \rightarrow trans}$ and $k_{trans \rightarrow cis}$ in unfolded $Trx1P_{ox}$. Assuming a $k_{cis \rightarrow trans}$:$k_{trans \rightarrow cis}$ ratio identical to the 95:5 ratio between fast and slow folders determined in

Supplementary Fig. 3d, values of $1.71 \pm 0.33 \cdot 10^{-2}$ s^{-1} for $k_{cis \rightarrow trans}$ and $8.99 \pm 4.47 \cdot 10^{-4}$ s^{-1} for $k_{trans \rightarrow cis}$ were obtained.

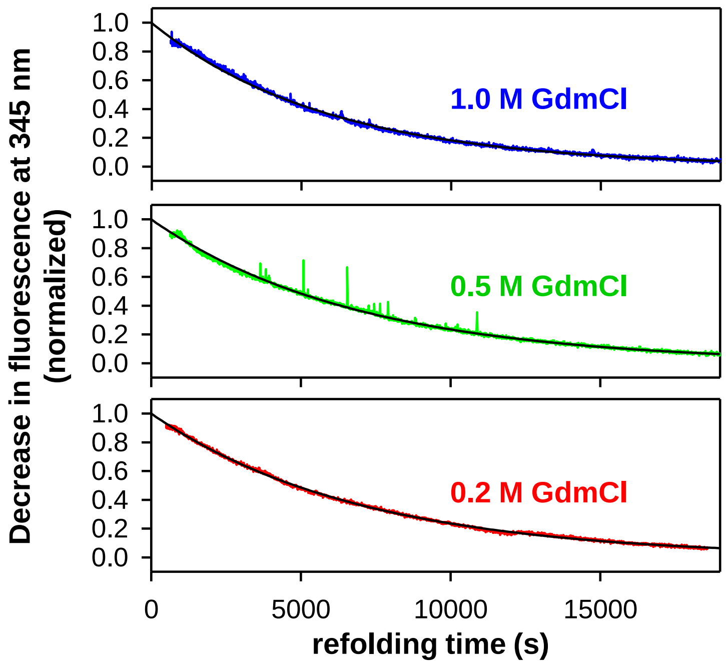

Supplementary Figure 6: The rate of the I_{trans}→N_{cis} transition during Trx1P_{ox} folding at 25 °C and pH 7.0 is independent of GdmCl concentration in the refolding buffer. The I_{trans}→N_{cis} reaction of Trx1P_{ox} (3 µM) in the presence of 0.2, 0.5 or 1.0 M GdmCl was recorded via the decrease in tryptophan fluorescence. The fluorescence traces were fitted mono-exponentially, yielding half-lifes of 68.1 ± 0.5 min, 79.3 ± 0.2 min and 79.9 ± 0.2 min for 0.2 M, 0.5 M and 1.0 M GdmCl, respectively.
